# Descending the Reconstruction Ladder: Single-Stage Full-Thickness Skin Grafting for Wide Nasal Skin Malignant Defects

**DOI:** 10.1055/s-0044-1801836

**Published:** 2025-02-24

**Authors:** Francisco J. Villegas-Alzate, Ana G. Cabezas-Charry, Víctor A. Cardona, Juan F. Ayala, José D. Villegas

**Affiliations:** 1 Plastic Surgery Unit, Clínica San Francisco, Tuluá, Valle, Colombia; 2 Department of Plastic Surgery, Universidad del Valle, Cali, Colombia; 3 Department of Statistics, Universidad San Martin, Cali, Colombia; 4 Department of Plastic Surgery,Universidad de Antioquia, Medellín, Colombia

**Keywords:** nose, skin, questionnaires, patient-reported outcome

## Abstract

**Background:**

Complex nasal reconstructions traditionally use staged flaps, with skin grafts reserved for smaller defects.

**Objective:**

This study evaluates single-stage full-thickness skin grafting (FTSG) for wide nasal defects postcancer resection.

**Materials and Methods:**

A retrospective analysis included 52 patients with nasal malignant lesions limited to the skin, reconstructed in a single stage immediately after cancer resections. Defects were intentionally over- or downsized to align with the esthetic unit concept. Templates of the defects were used to harvest FTSG. All donor areas were closed primarily. The graft was carefully sutured to fit the defect, and bolsters were applied for 5 to 12 days. Postoperative taping was used for 4 months. Postoperative photographs were assessed by 92 independent raters using a visual analog scale evaluating five parameters: skin color matching, surface regularity, symmetry, perimetral contours, and overall nasal appearance. Results and complications were analyzed for statistical associations.

**Results:**

On average, 3.5 of 9 nasal units per patient were reconstructed, covering 55.5% of the nasal surface. Local anesthesia was used in 90.4% of cases. Periclavicular and retroauricular donor sites were used in 61.5 and 34.6% of cases, respectively. Evaluators rated the outcomes at an average of 7.1/10 (range 5.1–8.8). The complication rate was 15.4%. No significant correlations were found between the outcomes and the analyzed factors.

**Conclusion:**

FTSG effectively reconstructs wide nasal defects in a single stage, predominantly under local anesthesia, with satisfactory outcomes. This approach signifies a descent down the reconstruction ladder, shifting from complex, flap-staged methods to a single-stage solution.

## Introduction

The nose is the body part most commonly affected by skin cancer. Immediate nose reconstruction exhibits unique challenges. 1 Depending on the defect, the reconstructive ladder includes varied methods such as healing by secondary intention, primary closure, delayed primary closure, skin grafting, compound grafting, local flaps, pedicled-staged forehead or auricular flaps, 2 3 4 and free flaps. 5 6 7 8

Traditionally, flaps are considered to offer superior cosmetic outcomes compared to skin grafts. 9 However, the use of local or regional flaps often necessitates additional procedures and results in facial scarring at the donor site. Skin grafts, on the other hand, are a simpler alternative suitable for small defects but are seldom employed for larger defects. 10

This study examines the use of full-thickness skin grafts (FTSGs) for the single-stage reconstruction of wide, limited-to-the-skin nasal defects, using the subunits approach. We detail the method, provide quantified external assessments of results, report complications, and explore correlations with variables including donor site, reconstruction extent, nasal area involvement, gender, Fitzpatrick's phototype, and age. This study also exposes, in more detail, our earlier brief communication on the topic. 11

## Materials and Methods

We conducted a retrospective review of medical records for 52 patients treated between June 2006 and June 2021. The study protocol was reviewed and approved by the Institutional Review Board (IRB) of Clínica San Francisco, Tuluá, ensuring adherence to ethical standards and patient safety.

### Inclusion Criteria

- Patients operated on by the senior author, F.J.V.-A.
- Nasal defects limited to the skin, resulting from skin cancer resection.
- Defects must preserve sufficient vascularized tissue, including perichondrium, periosteum, or nasal SMAS, to ensure successful FTSG adherence.
- In selected cases, deeper defects are included if adequate tissue mobilization provides a viable graft bed.
- Complete and assessable clinical records, pre- and postoperative photographs, with a follow-up period of more than 3 months.

### Exclusion Criteria

- Individuals undergoing flap-based nasal reconstruction.
- Defects extending beyond the skin into support structures that cannot be adequately prepared for grafting, even after local tissue mobilization.

### Procedure

Cancer resections were performed under magnification using ×2.5 loupes to ensure complete resection. The completeness and adequacy of the resection, as well as the decision to preserve a bed suitable for grafting, 12 13 were clinically assessed using loupe magnification and based on the preoperative histological diagnosis. No intraoperative margin confirmation was performed, but definitive histopathological margin confirmation was obtained in every case.

After resection, defects were intentionally oversized to align with the esthetic unit's concept. Nine esthetic units were considered for reconstruction and analysis: one dorsum, two side walls, one tip, two soft triangles, two alae, and one columella. 14

**Fig. 1:**
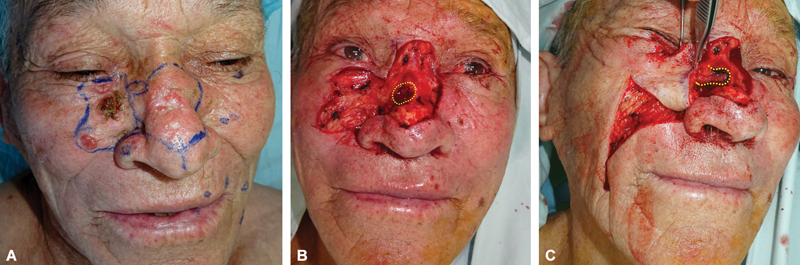
Wide nasal and cheek defect prepared for immediate skin graft reconstruction. ( **A** ) Facial and nasal nonmelanoma skin cancers with planned resections (blue lines). ( **B** ) Nasal defect postresection under local anesthesia, highlighting a transfixing defect (yellow-dotted circle) involving the sidewall and upper lateral cartilage. ( **C** ) Medially rotated paranasal flap filling the sidewall defect (yellow-dotted line); a cheek flap is adjusted for nasal subunit reconstruction.

**Fig. 2:**
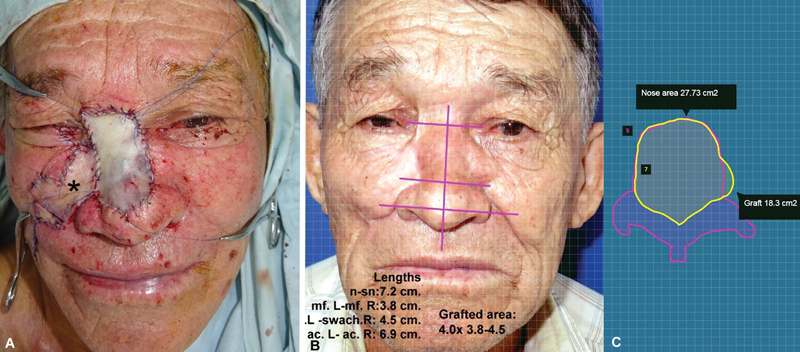
Same patient as in Fig. 1. ( **A** ) Intraoperative view of the right cheek flap (asterisk) and nasal subunits reconstructed with a full-thickness periclavicular skin graft before applying the over-tie dressing. ( **B** ) Late postoperative result. Software measurements show the grafted area as 66% of the nasal surface. External evaluators scored the result at 6.8/10 (0 = worst and 10 = best).

For defects larger than a single unit or exceeding the entire nose, raw areas were downsized to fit within the esthetic units, with neighboring tissues (e.g., cheek flaps). When the alar rim was involved, peninsular or propeller nasolabial flaps were employed to provide rim tissues and a vascularized bed for the skin graft. Cartilage grafts were not used for reinforcement in the alar rim or soft triangles. In cases where bone or cartilage was exposed during resection, adjacent nasal SMAS or subcutaneous flaps were used to cover the exposed framework, creating a vascularized bed to enhance graft integration 15 16 ( Figs. 1 and 2 ).

**Fig. 3:**
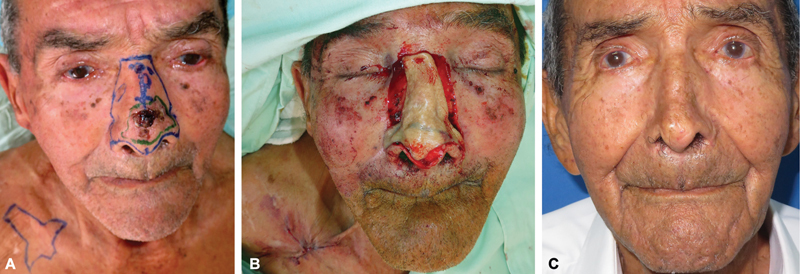
Nasal skin cancer resection and reconstruction. ( **A** ) Preoperative view showing multiple skin cancers on the nasal area; blue lines mark planned resection. Right periclavicular area selected as donor site. ( **B** ) Skin cancer resection performed under local anesthesia. Full-thickness skin graft harvested from right supraclavicular region and donor site closed. The graft must be sutured and immobilized. ( **C** ) Postoperative outcome.

Using templates (foil wrap of suture or the resected specimen), the donor area was marked. For harvesting the FTSG, we use the subdermal layer as the dissection plane. Residual fat, if present, was meticulously excised with scissors. Primary closure of each donor area was achieved. Minor defects were reconstructed with retroauricular skin, if direct closure of the donor area was possible, while larger areas and multiple subunits were treated with periclavicular skin. Following meticulous hemostasis in the nasal bed, the graft was correctly oriented and sutured to the nasal receptor area using a running suture with Polypropylene 5–0. Tie-over bolsters with antibiotic ointment were applied. The bolster was removed according to institutional logistics and patient accessibility to the service, between 5 and 12 days after surgery ( Fig. 3 ).

After the removal, dressing changes were recommended daily for 2 to 3 weeks and then replaced by taping for 4 months. The donor retroauricular area did not require specific postoperative treatment. In contrast, for the periclavicular area, we recommended applying porous adhesive tape every other day for a duration of 4 months. Clinical and photographic evaluations were performed at irregular intervals. Defects and total nasal areas were measured (cm ^2^ ). Anthropometric landmarks of the nose were determined: *n* , nasion; *sn* , subnasale; left and right *mf* , maxillofrontale points; and both *ac* : alar curvature points. Additionally, we defined bilateral points named *swach* : as the most lateral point of the alar crease that joins the cheek and sidewall esthetic units ( *s* : side, *w* : wall, *a* : alar, *c* : crease, *h* : hub). The nose perimeter was demarcated by connecting these points with lines. 17 18 19

**Fig. 4:**
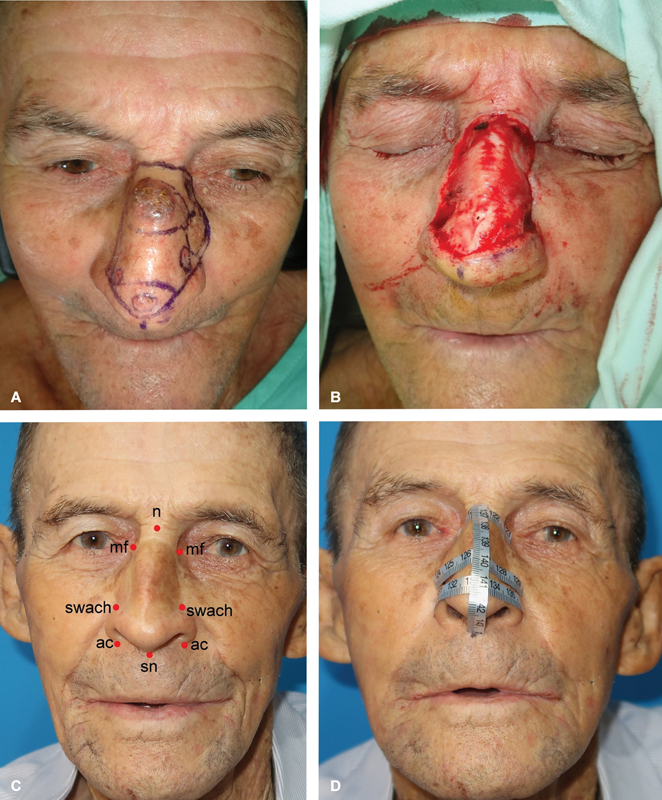
Nasal area measurements. Case 19, 73-year-old man. ( **A, B** ) Esthetic unit-based resection of confluent basal cell carcinomas under local anesthesia. ( **C** ) Postreconstruction, anthropometric landmarks: n (nasion), mf (maxillofrontale), swach (s: side, w: wall, a: alar, c: crease, h: hub), ac (alar curvature), sn (subnasale). ( **D** ) Adhesive measuring tape directly attached to nose for precise two-dimensional area calculation.

During postoperative photographic documentation, an adhesive measuring tape was directly applied to the skin to ascertain the actual dimensions of the nose and calculate the defect's size ( Fig. 4 ).

The total area of each treated nose, including reconstructed regions, along with their respective percentages, was computed using an online irregular area calculator (Sketch and calc). 20

Independent evaluation of outcomes followed the “visual analog scale” methodology, assessing postoperative photographs for graft-to-neighboring skin color matching, surface regularity or relief alignment, symmetry, perimetral contours or borderlines of the reconstructed area, and overall nasal appearance.

Each parameter was quantified using a 10-point linear scale, with “0” indicating the poorest outcome and “10” representing the best possible result. These assessments were conducted via Google Forms questionnaires, where sets of three full-face photographs of the results from 52 patients were uploaded.

Questionnaires were distributed via academic email lists and WhatsApp groups focused on plastic surgery, rhinoplasty, or dermatological surgery. Participation was voluntary, and raters were intentionally blinded to the reconstruction technique used, defect size, or complexity, as well as the identities of the authors and their respective institutions.

### Statistics

Statistical analysis was conducted to explore the relationships between independent raters' scores and various influencing factors, including affected mobile or fixed nasal units, the number of units involved, the percentage of the reconstructed nasal area, donor areas used, phototype, sex, and age.

To assess the normality of numerical variables and comparison groups, the Shapiro–Wilk's test was employed to determine the appropriate choice between parametric and nonparametric tests.

Correlations between variables were evaluated using either Spearman's or Pearson's correlation coefficients, depending on the distribution of the variables.

Group comparisons were conducted using the Student's *t* -test for normally distributed groups and the Mann–Whitney's *U* test for groups with nonnormally distributed data.

## Results

Fifty-two patients were enrolled in the study, with ages from 31 to 95 years. The study's average follow-up period was 42 months (3–168 months). Procedures were predominantly made under local anesthesia (47 patients, 90.4%), while the remaining 5 patients underwent procedures under general anesthesia.

The histological types of the resected specimens were basal cell carcinoma in 41 cases (78.9%), squamous cell carcinoma in 6 (11.5%), combined basal and squamous carcinoma in 3 (5.8%), and lentigo maligna melanoma in 2 cases (3.8%).

According to Fitzpatrick's phototypes, classification was as follows: type I in 3 cases (5.7%), II in 23 cases (44.2%), III in 22 cases (42.3%), and IV in 4 cases (7.7%), with no patients falling into the darker type V or VI.

The procedure was performed as the sole surgical intervention for nasal reconstruction in 94.2% of the cases (49 patients). Three patients required revisional surgeries (5.7%). Twenty-nine patients (55.7%) underwent associated procedures during the same resective and reconstructive surgery, including resection of other skin cancers, fulguration and curettage of premalignant lesions, and the use of local flaps to tailor defects to the dimensions of the nose or the boundaries of esthetic units.

The number of esthetic units reconstructed ranged from 1 to 8, with an average of 3.5 units per patient. In nine patients (17%), only one unit was reconstructed. The frequency of each reconstructed subunit is described in Table 1 .

**Table 1:** Patient and Surgery Characteristics with Quantified Outcomes: Single-Stage Skin Grafting for Extensive Nasal Defects

Demographics	Average or value (percentage/range)	
Age (y)	69.5 (31–95)	
30–39	1	
40–49	5	
50–59	7	
60–69	11	
70–79	18	
80–89	8	
≥90	2	
Females	39 (75%)	
Males	13 (25%)	
Surgery		
Local anesthesia	47 (90.4%)	
Clavicular skin graft	32 (61.5%).	
Retroauricular skin graft	18 (34.6%)	
Other skin grafts	2 (3.8%)	
One surgical procedure (resection reconstruction)	49 (94.2%)	
Two surgical procedures	3 (5.8%)	
Other associated procedures (i.e., other cancer resections or local flaps)	29 (55.7%)	
Histological type		
Basal cell carcinoma	41 (78.9%)	
Squamous cell carcinoma	6 (11.5%)	
Basal–squamous carcinoma	3 (5.8%)	
Lentigo maligna melanoma	2 (3.8%)	
Follow-up (mo)	42 (3–178)	
< 12	14 (44.2%)	
12–36	22 (42.3%)	
> 36	16 (30.7%)	
Phototype (Fitzpatrick)		
I	3 (5.7%)	
II	23 (44.2%)	
III	22 (42.3%)	
IV	4 (7.7%)	
V and VI	0	
Reconstructed nasal units average 3.5 (1–8)		
Dorsum	45 (86.5%)	
Right side wall	27 (52%)	
Left side wall	27 (52%)	
Tip	43 (82.7%)	
Right ala	12 (23%)	
Left ala	12 (23%)	
Right soft triangle	7 (13.4%)	
Left soft triangle	7 (13.4%)	
Columella	2 (13.8%)	
Nasal surface measurements		
Total nasal area (cm ^2^ )	25.4 (18.3–36.3)	
Reconstructed nasal area (%)	55.5 (12.2–97.3)	
Reconstructed nasal area (cm ^2^ )	14.1 range (3.3–30.4)	
Independent evaluators score (0–10 points): average 7.01 (range 5.1–8.8)		
Graft to neighboring skin color match	6.7	
Surface contour or relief matching	7.1	
Nasal symmetry	7.4	
Perimetral contours	6.6	
Global nasal appearance	7.2	

The average size of the nasal area was 25.4 cm ^2^ , with the reconstructed nasal areas covering an average of 55.5% of the total nasal surface area (range 12.2–97.5%).

Donor skin sources varied among the patients: periclavicular in 32 patients (61.5%), retroauricular skin grafts in 18 patients (34.6%), the proximal volar–ulnar forearm skin in 1 patient (1.9%) due to a preexisting skin lesion, and the groin skin in another patient who had multiple facial and extremity defects following cancer resection.

Ninety-two independent raters participated in the study and responded to the questionnaires. Each patient received from 29 to 31 scores, for each of the five evaluated parameters. Among the evaluators, 56 were plastic surgeons (61%), 9 were dermatologists (10%), 8 were ENT surgeons (9%), 5 were plastic surgery residents (5%), 3 were ENT residents (3%), and 11 were other medical professionals or specialists (12%).

The global average score was 7.1, with the worst being 5.1 and the best score being 8.8. No patient scored a perfect 10/10 (0 the worst and 10 the best) ( Table 1 ).

No statistically significant relationships were identified between quantified results or complications and variables such as donor site, extent of reconstruction, involvement of mobile or fixed nasal areas, gender, phototype, or age.

### Complications

The overall rate of complications was 15.4%. Partial graft loss was identified in five patients (9.6%) managed through healing by secondary intention. It is noteworthy that two out of these five patients had skin grafts placed on local subcutaneous flaps or nasal SMAS flaps (3.8%).

Additionally, two patients required further resection due to positive cancer margins. One patient experienced symptomatic scar retraction in the right inner canthal area, managed with excision and a Z-plasty procedure. Importantly, there was no residual tumor presence or cancer relapse within the grafted areas during the long-term follow-up period (up to 11 years). There were no cases of infection, wound dehiscence, or hematoma.

## Discussion

**Fig. 5:**
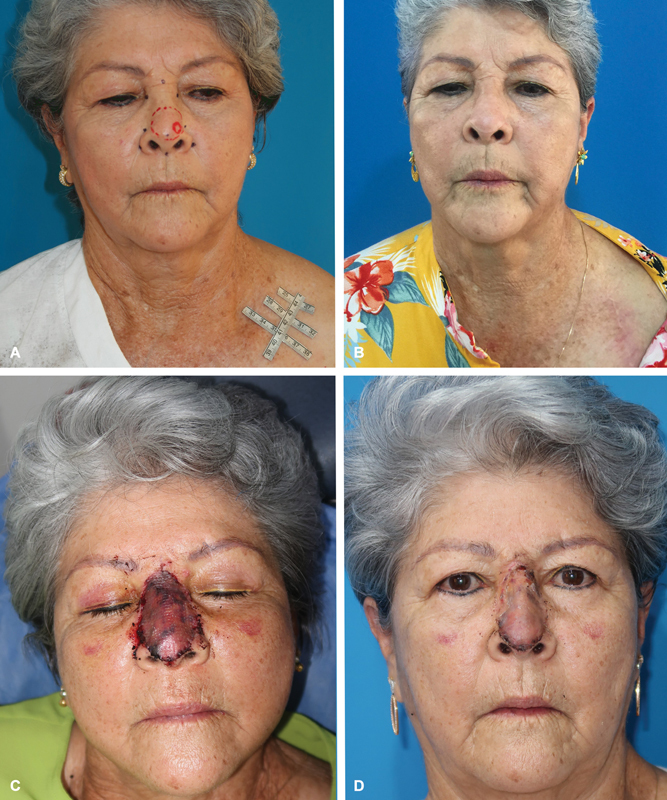
Nose skin graft evolution. ( **A** ) Preoperative area mapping with red (involved) and black (reference) dots. Left subclavicular skin selected as donor site. ( **B** ) Late postoperative picture after single-stage nasal reconstruction with full-thickness skin graft. ( **C** ) Five days postsurgery. Dressing removed, initial “graft take” is visible. Daily dressing changes are recommended. ( **D** ) Three weeks postsurgery. Complete graft integration with peripheral crusting. Skin emollients and porous taping are recommended for 4 months.

Our study shows FTSG can effectively reconstruct wide, nasal defects limited to the skin, traditionally managed with flaps. While the literature favors flaps for large defects, our results demonstrate that FTSG can achieve acceptable esthetic outcomes, as rated by independent evaluators. This expands on previous success with small defects. FTSG offers a simplified, single-stage alternative without facial donor-site morbidity, challenging the conventional reconstructive ladder approach ( Fig. 5 ). Our previous study using FACE-Q questionnaires showed high satisfaction and low appearance-related distress in 22 patients with wide nasal defects treated using this approach. 11

We found no statistically significant correlations between results or complications and variables such as donor site, reconstruction extent, nasal area type, gender, phototype, or age, likely due to consistently positive outcomes across our cohort.

Forehead flap reconstruction is optimal for the nose but requires secondary procedures, affecting patient quality of life and health care costs. Forehead and other interpolated flaps such as nasolabial also involve multiple procedures and facial donor scarring. 21 Compared to FTSG, local flaps have drawbacks such as larger scars crossing subunits, contour deformities, thickness issues, and trapdoor defects. Halani et al used FTSG for 36.2% of skin-only nasal defects, mainly in the upper two-thirds. However, FTSG is not suitable for exposed cartilage/bone and is challenging in the lower third due to shape, thick sebaceous skin, and alar retraction. 22 We propose extending graft-based reconstructions to wider skin-only defects, including challenging areas such as the tip, alae, and soft triangles.

Veldhuizen et al's systematic review of 176 articles involving 11,370 patients revealed that nasal skin defects larger than 1.5 cm were often reconstructed using the forehead flap, while smaller defects were managed with local flaps. The complication rate was 13.8%, and only a small fraction of articles (4.5%) reported patient satisfaction using standardized questionnaires. 23 In contrast, our study demonstrates that FTSGs can effectively reconstruct wide nasal defects in a single stage, supported by objective evaluations. This emphasizes skin grafts as a simpler alternative to the traditional forehead flap method.

Despite the disadvantages of delayed reconstruction, such as prolonged recovery, increased morbidity, and higher costs, the delay of FTSG and composite grafts in nasal reconstruction has been associated with certain advantages and fewer postoperative complications. Additionally, a staged approach allows for the option of “augmentation” of the skin graft using a dermal matrix, a perichondrocutaneous pseudo-composite graft, and staged structural grafting. 24 25 26 On the other hand, our series demonstrated the feasibility of single-stage surgery without compromising quality of life or increasing complications.

In our investigation, the overall rate of complications was 15.4%, which is similar to the findings of a study comparing flap and skin graft nasal reconstruction in 210 patients, where 41 individuals (19.5%) experienced postoperative problems. Interestingly, the overall complication rate did not significantly differ between the flap and graft groups. 27

Our study introduces a method for determining the nasal area, reconstructed area, and their proportion using cost-effective tools. We recommend adopting this method or transitioning to three-dimensional models and precise measurements for more standardized results in future studies. It is important to note that current anthropometric measurements often overlook nasal esthetic units, necessitating the creation of new landmarks by plastic surgeons. Our study introduces the “ *swach* ” landmark as a valuable addition to delineate the nasal perimeter more accurately.

The preference for the periclavicular donor area is due to its capacity to provide skin for complete nasal reconstruction while allowing for primary donor-site closure. For smaller defects, retroauricular skin is preferred for its ability to create a more discreet donor scar. In special circumstances, other areas can be used for nonperforating small nasal defects. A study suggests that for better skin match in small defects of the dorsum and sidewall, the preauricular area should be used, while conchal skin grafts are recommended for defects of the tip. However, these donor areas are insufficient for larger defects, as addressed in our current work. 28

Multiple actinic keratoses must be eliminated by lesion-directed therapies and field-directed therapies as the current treatment to prevent their probable progression into invasive squamous cell carcinoma. 29 While prophylactic surgery to reduce cancer risk has been adopted in the breast and other organs, surgical resection of premalignant skin lesions has received limited attention. 30 31 A single case report affords a debatable surgical resection of skin on the dorsum of the hand. 32

**Fig. 6:**
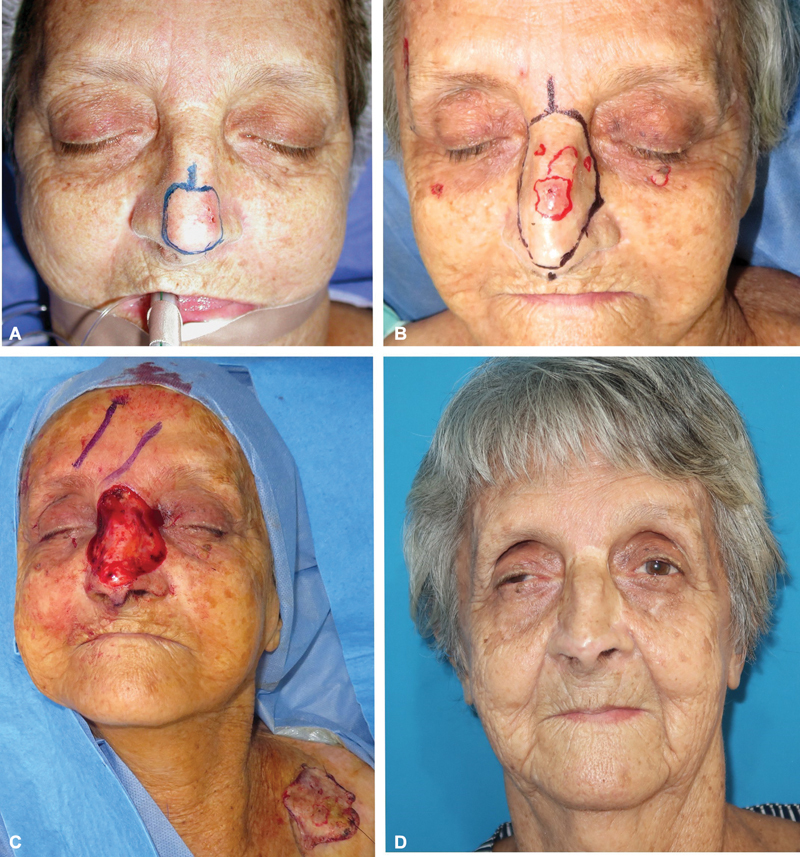
New cancer lesions 11 years after initial skin cancer removal. ( **A** ) At the age of 56 years, the patient underwent nasal tip unit resection with retroauricular skin graft reconstruction. ( **B** ) At the age of 67 years, additional resections on untreated nose. The initial graft remains unaffected. ( **C** ) Second wide nasal resection, with specimen used as a template for left supraclavicular skin graft. ( **D** ) Long-term follow-up after second resection. External ratings: 7.8/10. Based on cases like this, the authors propose the concept of “nose skin cancer prophylaxis with surgical resection and immediate reconstruction.”

Our long-term follow-up, extending up to 11 years, revealed that 11.5% of patients developed new lesions exclusively on nonresected nasal skin, while the grafted areas remained unaffected. This underscores the potential benefits of wider or complete nasal skin resection as a prophylactic measure in selected cases of premalignant or sun-damaged patients. This concept, termed “ *nose skin cancer prophylaxis with surgical resection and immediate reconstruction* ,” simplifies superficial nasal resections, resulting in positive outcomes and reducing the likelihood of repeated small resections, cancer recurrences, complex reconstructions, and poor results associated with conservative approaches ( Fig. 6 ).

**Fig. 7:**
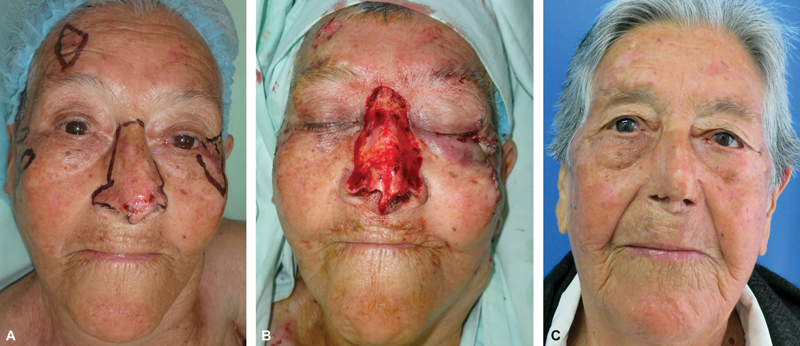
Single-stage wide nasal defect reconstruction. ( **A** ) Presurgical skin lesions with surgical guidelines, adaptable during the procedure. ( **B** ) Postresection view after subunit concept with noticeable nostril rim defect, under local anesthesia. A full-thickness clavicular skin graft was planned. ( **C** ) Postoperative result. External evaluation: 5.5/10 score reflects skin graft limitations: minimal dermal tissue, subsidence, retraction, and compromised alar rim contour. Future refinement procedures are recommended.

Reconstruction with FTSG for superficial wide defects of the nose has advantages such as local anesthesia procedure, no delays, single surgical time, no forehead scar, and more availability of donor areas. We acknowledge the disadvantages of skin grafts: lack of dermal tissue, they are not useful in cartilage or bone exposure and transfixing composite defects, they do not allow simultaneous framework reconstruction, and they have a typical subsidence deformity ( Fig. 7 ).

Assets of this study are the value of the number of independent ratings of results. Despite its merits, this study has certain limitations, including its retrospective nature, a limited number of cases, and reliance on a single surgeon's experience. Nevertheless, it offers the undeniable strength of many independent ratings.

## Conclusion

FTSG is an effective one-stage technique reconstruction, for wide nasal defects limited to the skin, offering advantages such as no facial donor scarring and the use of local anesthesia. We recommend extending its application to wide skin-only defects, including the challenging mobile nasal units. Based on long-term observations, we suggest “prophylactic” resection of damaged skin to prevent repeated procedures. FTSG proves reliable for extensive nasal surface defects, with favorable results supported by external ratings and patient-reported outcomes. This approach represents a descent down the reconstruction ladder, shifting from complex, staged methods to a single-stage solution for wide nasal defects. 11

## References

[1] Veldhuizen I J Brouwer P Aleisa A Nasal skin reconstruction: time to rethink the reconstructive ladder? J Plast Reconstr Aesthet Surg 2022 75 03 1239 1245 34903490 10.1016/j.bjps.2021.11.028 PMC8976754

[2] Washio H Retroauricular-temporal flap Plast Reconstr Surg 1969 43 02 162 166 4885510 10.1097/00006534-196902000-00009

[3] Orticochea M A postauricular flap to reconstruct facial defects Br J Plast Surg 1976 29 04 325 333 793665 10.1016/0007-1226(76)90016-3

[4] Galvao M S A postauricular flap based on the contralateral superficial temporal vessels Plast Reconstr Surg 1981 68 06 891 897 6272340 10.1097/00006534-198112000-00008

[5] Han Y Pan Y Yang L Resurfacing partial nose defects with a retroauricular skin/cartilage free flap Ann Plast Surg 2011 67 01 34 39 21102309 10.1097/SAP.0b013e3181d50e80

[6] Spataro E Branham G H Principles of nasal reconstruction Facial Plast Surg 2017 33 01 9 16 28226366 10.1055/s-0036-1597949

[7] Joseph A W Truesdale C Baker S R Reconstruction of the nose Facial Plast Surg Clin North Am 2019 27 01 43 54 30420072 10.1016/j.fsc.2018.08.006

[8] Gasteratos K Spyropoulou G A Chaiyasate K Microvascular reconstruction of complex nasal defects: case reports and review of the literature Plast Reconstr Surg Glob Open 2020 8 07 e3003 32802686 10.1097/GOX.0000000000003003 PMC7413796

[9] Jacobs M A Christenson L J Weaver A L Clinical outcome of cutaneous flaps versus full-thickness skin grafts after Mohs surgery on the nose Dermatol Surg 2010 36 01 23 30 19889165 10.1111/j.1524-4725.2009.01360.x

[10] Sapthavee A Munaretto N Toriumi D M Skin grafts vs local flaps for reconstruction of nasal defects: a retrospective cohort study JAMA Facial Plast Surg 2015 17 04 270 273 26021837 10.1001/jamafacial.2015.0444

[11] Villegas-Alzate F J Cabezas-Charry A G Cardona V A Ayala J F Villegas J D Single-stage reconstruction of very-wide nasal defects with full-thickness skin grafts: retrospective analysis of patient reported outcomes J Plast Reconstr Aesthet Surg 2024 93 100 102 38678811 10.1016/j.bjps.2024.04.027

[12] Ozturk C N Larson J D Ozturk C Zins J E The SMAS and fat compartments of the nose: an anatomical study Aesthetic Plast Surg 2013 37 01 11 15 23296757 10.1007/s00266-012-0012-1

[13] Neves J C Zholtikov V Cakir B Coşkun E Arancibia-Tagle D Rhinoplasty dissection planes (subcutaneous, sub-SMAS, supra-perichondral, and sub-perichondral) and soft tissues management Facial Plast Surg 2021 37 01 2 11 33634451 10.1055/s-0041-1723825

[14] Burget G C Menick F J The subunit principle in nasal reconstruction Plast Reconstr Surg 1985 76 02 239 247 4023097 10.1097/00006534-198508000-00010

[15] Lindsay K J Morton J D Flap or graft: the best of both in nasal ala reconstruction J Plast Reconstr Aesthet Surg 2015 68 10 1352 1357 26188401 10.1016/j.bjps.2015.05.036

[16] Xu M Yang C Wang W J Bi H D Xing X An “oxhorn”-shaped V-Y advancement flap unilaterally pedicled on a nasal superficial musculoaponeurotic system for nasal reconstruction J Plast Reconstr Aesthet Surg 2015 68 11 1516 1521 26243194 10.1016/j.bjps.2015.07.016

[17] Farkas L G Kolar J C Munro I R Geography of the nose: a morphometric study Aesthetic Plast Surg 1986 10 04 191 223 3812136 10.1007/BF01575292

[18] Mehta N Srivastava R K The Indian nose: an anthropometric analysis J Plast Reconstr Aesthet Surg 2017 70 10 1472 1482 28729079 10.1016/j.bjps.2017.05.042

[19] Celikoyar M M Pérez M F Akbaş M I Topsakal O Facial surface anthropometric features and measurements with an emphasis on rhinoplasty Aesthet Surg J 2022 42 02 133 148 33855336 10.1093/asj/sjab190

[20] iCalc: Irregular area calculator SketchAndCalc. Accessed February 6, 2022 at:https://www.sketchandcalc.com

[21] Sanniec K Malafa M Thornton J F Simplifying the forehead flap for nasal reconstruction: a review of 420 consecutive cases Plast Reconstr Surg 2017 140 02 371 380 28376026 10.1097/PRS.0000000000003540

[22] Halani S H Ma C Pierce J Sanniec K Thornton J F Nasal reconstruction after Mohs cancer resection: lessons learned from 2553 consecutive cases Plast Reconstr Surg 2021 148 01 171 182 34181615 10.1097/PRS.0000000000008098

[23] Veldhuizen I J Budo J Kallen E JJ A systematic review and overview of flap reconstructive techniques for nasal skin defects Facial Plast Surg Aesthet Med 2021 23 06 476 481 33650884 10.1089/fpsam.2020.0533 PMC10027346

[24] David A P Miller M Q Park S S Christophel J J Comparison of outcomes of early vs delayed graft reconstruction of Mohs micrographic surgery defects JAMA Facial Plast Surg 2019 21 02 89 94 30422211 10.1001/jamafacial.2018.1204 PMC6439791

[25] Robinson J K Dillig G The advantages of delayed nasal full-thickness skin grafting after Mohs micrographic surgery Dermatol Surg 2002 28 09 845 851 12269881 10.1046/j.1524-4725.2002.02031.x

[26] Landeen K C Davis S J Dedhia R D Shastri K S Ries W R Stephan S J Augmented skin grafting: a new rung in the reconstructive ladder Facial Plast Surg Aesthet Med 2022 24 02 126 129 34780298 10.1089/fpsam.2021.0112

[27] Rustemeyer J Günther L Bremerich A Complications after nasal skin repair with local flaps and full-thickness skin grafts and implications of patients' contentment Oral Maxillofac Surg 2009 13 01 15 19 18936990 10.1007/s10006-008-0139-z

[28] Gloster H M Jr The use of full-thickness skin grafts to repair nonperforating nasal defects J Am Acad Dermatol 2000 42 06 1041 1050 10827411

[29] Dianzani C Conforti C Giuffrida R Current therapies for actinic keratosis Int J Dermatol 2020 59 06 677 684 32012240 10.1111/ijd.14767

[30] Alaofi R K Nassif M O Al-Hajeili M R Prophylactic mastectomy for the prevention of breast cancer: review of the literature Avicenna J Med 2018 8 03 67 77 30090744 10.4103/ajm.AJM_21_18 PMC6057165

[31] Noguès C Mouret-Fourme E [Prophylactic surgery in common hereditary cancer syndromes] Bull Acad Natl Med 2012 196 07 1237 1245 23815011

[32] Hessam S Georgas D Sand M Bechara F G Complete skin resection of the dorsum of the hand: a prophylactic approach using a dermal regeneration template J Cutan Med Surg 2014 18 01 56 59 24377475 10.2310/7750.2013.13061

